# HIV Transgenic Rats Demonstrate Impaired Sensorimotor Gating But Are Insensitive to Cannabinoid (Δ9-Tetrahydrocannabinol)-Induced Deficits

**DOI:** 10.1093/ijnp/pyab053

**Published:** 2021-08-02

**Authors:** Benjamin Z Roberts, Arpi Minassian, Adam L Halberstadt, Yinong V He, Muhammad Chatha, Mark A Geyer, Igor Grant, Jared W Young

**Affiliations:** 1 Department of Psychiatry, University of California, San Diego, California, USA; 2 VA Center of Excellence for Stress and Mental Health, Veterans Administration San Diego HealthCare System, San Diego, California, USA; 3 VISN-22 Mental Illness Research Education and Clinical Center, VA San Diego Healthcare System, San Diego, California, USA

**Keywords:** CBD, HAND, HIV, PPI, THC

## Abstract

**Background:**

HIV-associated neurocognitive disorder (HAND) is commonly observed in persons living with HIV (PWH) and is characterized by cognitive deficits implicating disruptions of fronto-striatal neurocircuitry. Such circuitry is also susceptible to alteration by cannabis and other drugs of abuse. PWH use cannabis at much higher rates than the general population, thus prioritizing the characterization of any interactions between HIV and cannabinoids on cognitively relevant systems. Prepulse inhibition (PPI) of the startle response, the process by which the motor response to a startling stimulus is attenuated by perception of a preceding non-startling stimulus, is an operational assay of fronto-striatal circuit integrity that is translatable across species. PPI is reduced in PWH. The HIV transgenic (HIVtg) rat model of HIV infection mimics numerous aspects of HAND, although to date the PPI deficit observed in PWH has yet to be fully recreated in animals.

**Methods:**

PPI was measured in male and female HIVtg rats and wild-type controls following acute, nonconcurrent treatment with the primary constituents of cannabis: Δ ^9^-tetrahydrocannabinol (THC; 1 and 3 mg/kg, s.c.) and cannabidiol (1, 10, and 30 mg/kg, i.p.).

**Results:**

HIVtg rats exhibited a significant PPI deficit relative to wild-type controls. THC reduced PPI in controls but not HIVtg rats. Cannabidiol exerted only minor, genotype-independent effects on PPI.

**Conclusions:**

HIVtg rats exhibit a relative insensitivity to the deleterious effects of THC on the fronto-striatal function reflected by PPI, which may partially explain the higher rates of cannabis use among PWH.

**Significance Statement**

HIV-associated neurocognitive disorder (HAND) is a syndrome of cognitive impairments that limits HIV patients’ ability to function day-to-day. Concomitant chronic exposure to HIV proteins and cannabis produces greater cognitive deficits than either condition alone, although cannabis may also exert therapeutic effects in this population. Study of the effects of the chemical constituents of cannabis on cognitively relevant neurocircuitry in an animal model of HIV/HAND, the HIV transgenic (HIVtg) rat, may help to disentangle the individual contributions of cannabinoid compounds to the deleterious vs beneficial effects of cannabis. The present study found that prepulse inhibition, an assay of cognitively relevant neurocircuit integrity deficient in HIVtg rats and HIV patients, was not disrupted by the primary psychoactive constituent of cannabis, Δ9-tetrahydrocannabinol (THC), at doses sufficient to produce deficits in control animals. These results suggest a relative resistance to the deleterious effects of THC in the HIV disease state.

## Introduction

The implementation of combination antiretroviral therapy (cART) has reduced the mortality rate associated with Human Immunodeficiency Virus (HIV) such that persons living with HIV (PWH) are able to live full lives (Wada et al., 2013). This increased survivability necessitates the study of HIV as a chronic disease, the effective long-term management of which requires the consideration of comorbid disorders. Substance abuse, for example, is observed in this population at rates approaching 50% in the United States (Turner et al., 2001; Hartzler et al., 2017). Cannabis in particular is used by PWH at far higher rates than the general population (27%–32% vs 13%; Turner et al., 2001; Compton et al., 2016; Pacek et al., 2018). In addition to recreational use, PWH commonly use cannabis for purposes of self-medication and symptom management, citing its antiemetic, appetite-stimulating, and mood-enhancing effects (Dansak, 1997; Fairfield et al., 1998). Nevertheless, HIV^+^ cannabis users have elevated detectable viral loads relative to drug-abstinent PWH (Lee et al., 2020).

Another complication in the management of HIV is HIV-associated neurocognitive disorder (HAND), a syndrome of mild to moderate impairments in learning, memory, and executive function that affects at least 40% of all PWH in the United States (Heaton et al., 2010; Heaton et al., 2011). This pattern of deficits implicates pathology of fronto-striatal circuitry (Sahakian et al., 1995; Cole et al., 2007), which is also affected by cannabis (Martin-Santos et al., 2010; Ma et al., 2018). Indeed, a growing body of evidence indicates that the drug’s detrimental cognitive effects (e.g., Ranganathan and D’Souza, 2006; Crean et al., 2011) are more severe in PWH than in healthy populations (Skalski et al., 2016; Thames et al., 2016, 2017); however, other studies have identified anti-inflammatory and antiemetic effects of cannabis that may forestall the progression of HAND by counteracting the effects of HIV proteins and facilitating medication adherence (de Jong et al., 2005; Manuzak et al., 2018). Further investigation of the effects of concurrent cannabis use and HIV protein expression on cognition-relevant neurocircuitry and behaviors is therefore warranted.

**Figure 1.**
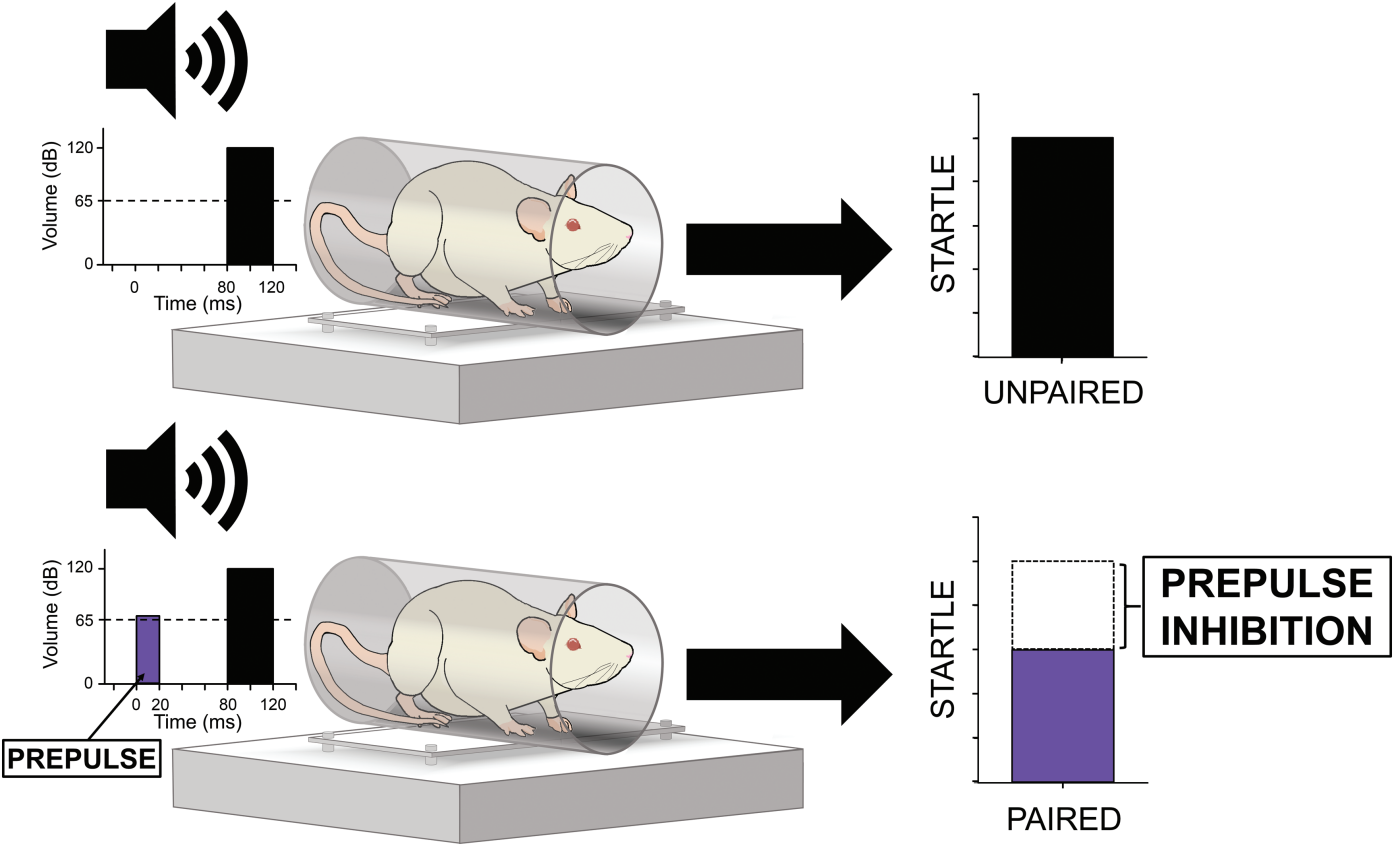
Prepulse inhibition (PPI) of the acoustic startle response (ASR). PPI is the process by which the ASR to a sudden high-intensity stimulus (top) is substantially reduced when that stimulus is immediately preceded by a much lower intensity, non-startling stimulus (bottom). Stimulus onset and magnitude of these two conditions is indicated at left, with background noise levels represented by horizontal dotted lines. Response magnitude is indicated at right (arbitrary units). PPI is reported as percent decrement of the ASR and is calculated using the average ASR magnitudes elicited by prepulse-paired (bottom) and unpaired (top) high intensity stimuli.

Also subserved by fronto-striatal circuitry is sensorimotor gating, the pre-attentive process mediating the suppression of motor responses to irrelevant sensory stimuli (Swerdlow et al., 2001). Prepulse inhibition (PPI), the attenuation of the startle response to a sudden high-intensity stimulus by the perception of a preceding non-startling stimulus (Figure 1), is an operational measure of sensorimotor gating that is deficient in a range of neurological disorders, including HIV/HAND (Minassian et al., 2013, Walter et al., 2021). While certainly not a proxy for cognition per se, PPI can be used to probe the integrity of descending forebrain circuitry that may be implicated in psychopathology-related cognitive dysfunction (Swerdlow et al., 2016). Baseline PPI correlates with certain measures of cognition and/or functional competence to a greater or lesser degree within individual patient populations—for example, attention and processing speed in Parkinson’s disease (Zoetmulder et al., 2014), general intelligence in Fragile X syndrome (Hessl et al., 2009), and functional status, but not cognition or symptom severity, in schizophrenia (Swerdlow et al., 2006). Studies of sensorimotor gating in PWH provide evidence for such a correlation in the HIV disease state. One of 2 such studies of which we are aware identified PPI deficits in only those PWH meeting criteria for HAND (Minassian et al., 2013), while the second revealed significant correlations between PPI and learning, memory, motor function, and global deficit scores in a much larger cohort of HIV^+^ males (although the specificity of PPI deficits to HAND was not replicated) (Walter et al., 2021). While these correlations by no means guarantee shared etiology between these 2 classes of deficits (disruptions of any one or combination of nuclei within the startle circuit may decrease PPI; Swerdlow et al., 2008), they may indicate some degree of overlap between the circuit-level pathology underlying HIV-mediated PPI deficiency and that effecting the pattern of neurocognitive impairment specific to HAND. Given that cannabis reduces PPI (Morales-Muñoz et al., 2017), it is important to determine any interactions of HIV and cannabinoids on this and other measures of fronto-striatal integrity. The cross-species validity of PPI (Swerdlow et al., 2008) provides an opportunity to study such interactions via assessment of the startle response in model organisms.

The Fischer 344 HIV transgenic (HIVtg) rat constitutively expresses 7 of the 9 genes that comprise the HIV genome and is a commonly used model for the cognitive and behavioral effects of chronic, non-replicative HIV infection (Reid et al., 2001; Vigorito et al., 2015). Previous studies report that HIVtg rats exhibit intact PPI relative to wild-type (WT) controls (Moran et al., 2013b) as well as generally lower startle reactivity to prepulse-preceded stimuli as measured by mean peak amplitude of the startle response (McLaurin et al., 2017a, 2017b, 2017c, 2018a; but see Moran et al., 2012; Roscoe et al., 2014). Consistent across these reports, however, is a temporal processing deficit whereby HIVtg rats demonstrate reduced sensitivity to manipulations of inter-stimulus interval duration (Moran et al., 2013a; McLaurin et al., 2016, 2017b, 2017c, 2019b) and/or shifts in temporal window for peak PPI (Moran et al., 2013a, 2013b; McLaurin et al., 2018a; but see Moran et al., 2012; Roscoe et al., 2014). These alterations may reflect disrupted dopamine dynamics, a well-established trait of the HIVtg rat (Moran et al., 2012, 2013b). Indeed, dopamine-relevant synaptic dysfunction has been observed in the HIVtg nucleus accumbens (Roscoe et al., 2014; Javadi-Paydar et al., 2017; McLaurin et al., 2018c), a key structure in PPI regulation (Swerdlow et al., 1992). To our knowledge no studies to date have examined the effects of cannabis on the startle response of the HIVtg rat. It is important to note, however, that cannabis is not a unitary agent—its primary constituents, Δ9-tetrahydrocannabinol (THC) and cannabidiol (CBD), have very different pharmacological profiles and subjective effects. Determining the impact of these 2 cannabinoids on PPI may inform the development of novel treatments that produce the therapeutic effects reported by self-medicating PWH while avoiding the negative consequences of cannabis use on those fronto-striatal functions assayed by PPI.

THC, the primary psychoactive component of cannabis, is a partial agonist of CB1 and CB2 cannabinoid receptors (Pertwee, 2008). CB1 receptors are found in several major nuclei of murine PPI regulatory circuitry (Swerdlow et al., 1992)—for example, nucleus accumbens, ventral tegmental area, hippocampus, and frontal cortex (Herkenham et al., 1991; Robbe et al., 2001)—while CB2 receptors are expressed primarily in immune cells (Pertwee, 2008). THC increases dopamine release and signaling in the rat nucleus accumbens (Tanda et al., 1997), which, given the striatal dopamine dysregulation characteristic of HIVtg rats, may normalize any accompanying PPI deficits. Past studies of the effects of THC on PPI in healthy (WT) rodents have yielded mixed results—THC produced PPI reductions in rats (Tournier and Ginovart, 2014) and mice (Nagai et al., 2006) in some studies, but not in others (Malone and Taylor, 2006; Long et al., 2010; Hložek et al., 2017). The behavioral profile of THC may therefore be influenced by such variables as rat/mouse strain, route of administration, and chronicity of treatment. These inconsistencies, as well as the fact that none of these investigations used Fischer 344 rats, the HIVtg parent strain, make it difficult to predict the effects of THC on sensorimotor gating in the HIVtg rat. Nevertheless, given the altered dopamine dynamics of HIVtg rats, it was predicted that THC would normalize any PPI deficits.

Meanwhile, CBD, the major non-psychoactive component of cannabis (Pertwee, 2008), reduced PPI in WT rats in some studies (Gururajan et al., 2011; Hložek et al., 2017) and increased PPI in others (Levin et al., 2014). The effects of CBD, like those of THC, may therefore also be influenced by rat strain and route of administration. CBD generally increases PPI in mice, however (Long et al., 2010; but see Long et al., 2006), and reliably rescues PPI deficits in putative animal models of schizophrenia (Long et al., 2006; Gomes et al., 2014; Levin et al., 2014; Peres et al., 2018), thereby demonstrating antipsychotic-like activity consistent with its effects in humans (Bhattacharyya et al., 2010). It was therefore hypothesized that, like THC, CBD would also attenuate any PPI deficits arising from dopamine dysregulation in the HIVtg rat.

Given the high rates of cannabis use among PWH, it remains imperative to determine the impact of the primary constituents of cannabis on cognition-relevant behaviors in model systems. We hypothesized that (1) HIVtg rats would exhibit deficient PPI relative to WT controls, (2) acute THC and CBD would produce differential effects on rats in a genotype-dependent manner, and (3) the directions of these effects would reveal normalization of HIV-mediated PPI deficits by both THC and CBD.

## MATERIALS AND METHODS

### Animals

Male and female HIVtg and WT Fischer 344 rats were used in the present study (8/group; Envigo; Indianapolis, IN, USA). Rats were housed in dyads in transparent plastic chambers with food and water available ad libitum. All rats were housed in a climate-controlled room on a 12-h-light/-dark schedule (7:00 am-7:00 pm dark). Testing was conducted during the dark portion of rats’ photoperiods and commenced when rats were 15 weeks of age. Rats were maintained in a dedicated animal facility compliant with all federal and state requirements and approved by the American Association for Accreditation of Laboratory Animal Care.

### Apparatus/Basic Startle Procedure

Acoustic startle response (ASR) was assessed using startle chambers consisting of cylindrical, non-restrictive Plexiglas stabilimeters (8.2-cm diameter) housed in illuminated, sound-attenuating cabinets (SR-LAB System, San Diego Instruments, San Diego, CA, USA). Speakers were mounted 24 cm above the stabilimeters. Startle amplitude was detected via piezoelectric accelerometer and transmitted digitally to a PC. Startle response was quantified as the average startle amplitude across the 100-millisecond recording window beginning at stimulus onset. Stabilimeters were calibrated and sound levels measured as described previously (Mansbach et al., 1988).

Testing sessions comprised the following: (1) pulse-alone trials, during which a single 40-millisecond 120-dB pulse of broadband white noise was delivered; and (2) prepulse trials, during which a 20- millisecond prepulse of considerably lower intensity (68, 71, or 77 dB) was delivered 100 millisecond prior to a 40-millisecond 120-dB pulse. A constant 65-dB broadband noise was present in the background during testing sessions. Main testing phases comprised 14 pulse-alone trials and 36 prepulse trials (12 of each prepulse intensity) distributed pseudorandomly. Main testing sessions were bracketed by “habituation” blocks of 5 pulse-alone trials (HABIT1 and HABIT2, respectively). The inter-trial interval varied between 9 and 21 seconds. Startle sessions were preceded by a 5-minute acclimation period. Startle was primarily quantified via the following measures:

1. Mean peak amplitude of ASR recorded during pulse-alone trials (excluding HABIT1/2 trials). Reported in arbitrary units.
2. PPI, the percent decrement of ASR recorded in prepulse trials vs pulse-alone trials (excluding HABIT1/2 trials; Figure 1). Calculated following the formula:

$$\begin{array}{l} \left[100-\left(\frac{Startle\;Amplitude_{Prepulse\;Trials}}{Startle\;Amplitude_{Pulse-alone\;Trials}\;\;\;}\times 100\right)\right] \end{array}$$

Secondary outcome measures are described in supplementary Material.

### Experimental Design

**Figure 2.**
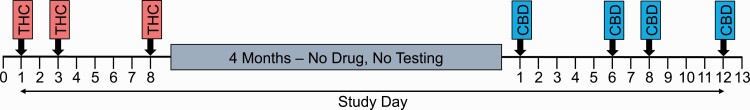
Study timeline. THC=Δ9-tetrahydrocannabinol (THC) assessment, during which rats were assessed in the 30-minute startle paradigm 30 minutes following subcutaneous administration of vehicle or 1 or 3 mg/kg THC; CBD=cannabidiol (CBD) assessment, during which rats were assessed in the same 30-minute startle paradigm 30 minutes following intraperitoneal administration of vehicle or 1, 10, or 30 mg/kg CBD. No injections or startle assessments were administered on days other than those indicated by arrows.

Rats aged 15 weeks (HIVtg: males: 245–300 g; females: 160–200 g; WT: males: 270–350 g; females: 160–200 g) were treated with subcutaneous THC (0, 1, and 3 mg/kg) and were assessed in the startle session described above following a within-subjects design (Figure 2). After a washout period of 4 months, the same rats (now aged 36 weeks) (HIVtg: males: 300–364 g; females: 185–205 g; WT: males: 330–470 g; females: 195–230 g) received i.p. CBD (0, 1, 10, and 30 mg/kg) and were tested in the same startle paradigm following a similar within-subjects design (Figure 2). In both studies, rats were injected 30 minutes prior to being placed in startle chambers. Injection volume was 1 mL/kg body weight. Dosage order was counterbalanced across rats.

### Drug Preparation

A 5-mg/mL solution of THC dissolved in ethanol was obtained from the National Institute on Drug Abuse. The ethanol was evaporated under a stream of dry nitrogen, and the residue was dissolved to final concentrations of 1 and 3 mg/mL in a vehicle consisting of 7.5% Tween-80 and 7.5% propylene glycol (Sigma-Aldrich, St. Louis, MO, USA) in saline. CBD (1, 10, and 30 mg/mL; Cayman Pharma, Prague, Czech Republic) was dissolved in a vehicle consisting of 6% polyethylene glycol and 50% (2-hydroxypropyl)-β-cyclodextrin (Sigma-Aldrich) in water.

### Statistical Analyses

PPI data from the 2 assessments were analyzed via 4-factor ANOVA using drug dose (THC or CBD) and prepulse intensity as within-subjects factors, and sex and genotype as between-subjects factors. ASR data were analyzed via 3-factor ANOVA using dose as a within-subject factor and sex and genotype as between-subjects factors. Given that it was hypothesized a priori that HIVtg rats would demonstrate significantly lower PPI than WT controls in the absence of any pharmacological manipulation, planned repeated-measures ANOVAs were conducted on vehicle data alone using genotype and sex as between-subjects factors and prepulse intensity as a within-subjects factor. To test the a priori hypothesis that THC and CBD would differentially affect HIVtg- vs WT rats, further planned ANOVAs were conducted on data from the 2 genotypes separately using dose and prepulse intensity as within-subjects factors and sex as a between-subjects factor. Significant (*P *< .05) and trend-level (*P < *.10) interactions were investigated further via follow-up ANOVAs or Tukey post hoc comparisons. Effect sizes for all ANOVAs were estimated via partial η ^2^. Data were analyzed using SPSS 24.0 (Chicago, IL, USA).

## RESULTS

### THC Assessment

**Figure 3.**
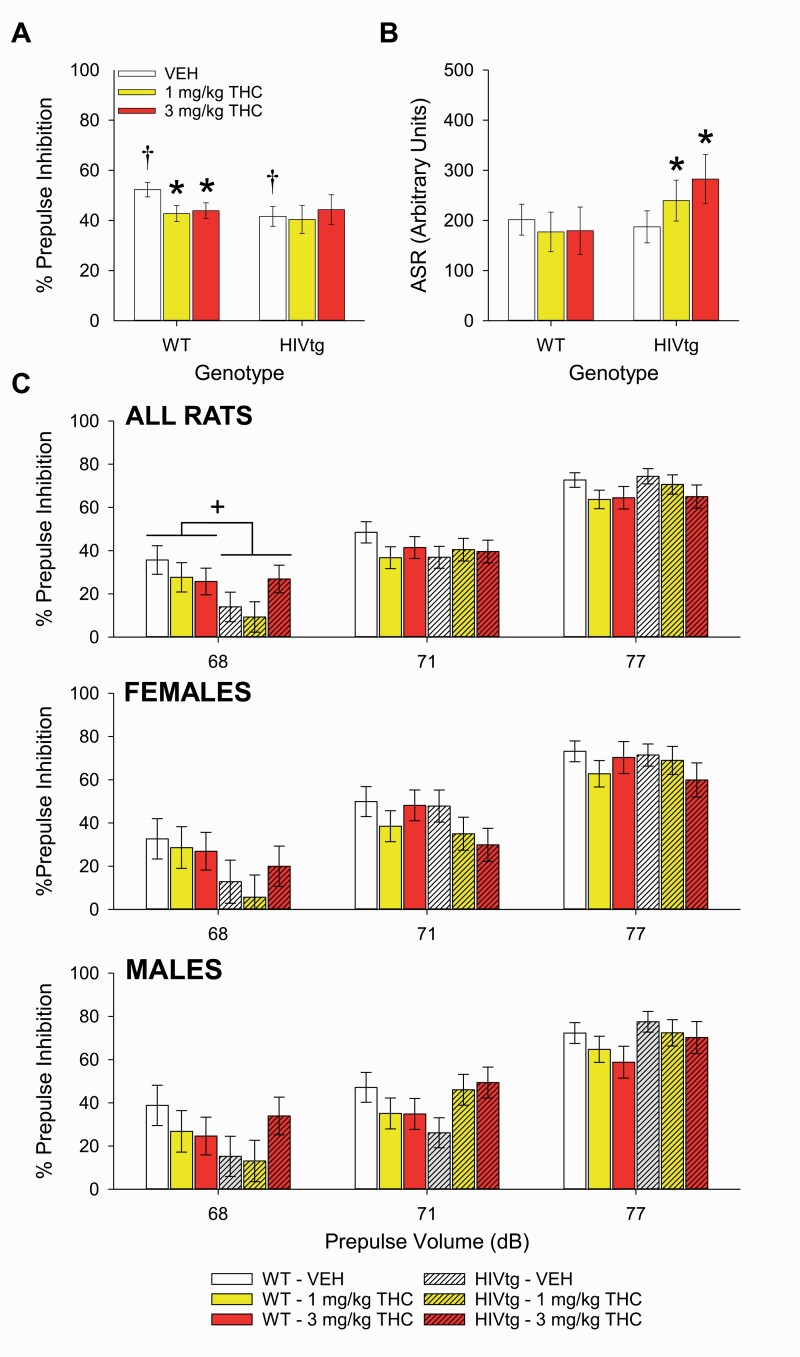
The effects of Δ9-tetrahydrocannabinol (THC) on prepulse inhibition (PPI) of the acoustic startle response (ASR) in male and female HIV transgenic (HIVtg) and wild-type (WT) rats. HIVtg rats displayed lower levels of PPI (averaged across prepulse intensities) following vehicle administration than WT rats. THC lowered PPI in WT rats only and did not affect HIVtg rats on this measure (A). THC affected ASR in HIVtg rats only, with both 1 and 3 mg/kg THC increasing ASR relative to vehicle (B). Follow-up analysis of a significant prepulse × genotype interaction revealed that HIVtg rats exhibited lower levels of PPI following 68-dB prepulses only (C) (top) regardless of sex (middle, bottom). ^+^*P* < .05; *P < .05 vs vehicle; bars annotated with † significantly differ from each other (*P* < .05). Data presented as mean ± SEM.

No main effects of THC (F_[2,54]_ = 1.2, *P* = .3, η _p_^2^ = 0.043), genotype (F_[1,27]_ = 0.3, *P* = .3, η _p_^2^ = 0.036), or sex (F_[1,27]_ = 0.1, *P* = .8, η _p_^2^ = 0.004) were observed on overall PPI, although a main effect of prepulse intensity was detected (F_[2,108]_ = 165.5, *P < *.001, η _p_^2^ = 0.86) whereby progressively louder prepulses elicited greater inhibition (all *P < *.001). Follow-up analysis of a prepulse × genotype interaction (F_[2,108]_ = 165.5, *P < *.01, η _p_^2^ = 0.16) revealed that HIVtg rats demonstrated reduced PPI during 68-dB prepulse conditions (F_[1,27]_ = 7.6, *P < *.05, η _p_^2^ = 0.22; Figure 3C, top). A priori planned analysis of vehicle data alone revealed that vehicle-treated HIVtg rats exhibited deficient PPI relative to vehicle-treated WT rats (F_[1,27]_ = 4.4, *P < *.05, η _p_^2^ = 0.14; Figure 3A), with a near-significant trend toward prepulse × genotype interaction (F_[2,54]_ = 3.1, *P* = .052, η _p_^2^ = 0.10) suggesting a specificity to 68-dB prepulse conditions (F_[1,27]_ = 5.2, *P < *.05, η _p_^2^ = 0.16). Further planned analyses of WT and HIVtg rats separately revealed that THC lowered PPI in WT rats (F_[2,28]_ = 4.0, *P < *.05, η _p_^2^ = 0.22), but not HIVtg rats (F_[2,26]_ = 0.2, *P* = .8, η _p_^2^ = 0.01; Figure 3A). No main or interactive effects of sex were observed during any of the above planned analyses.

THC did not independently affect ASR to unpaired pulses (F_[2,54]_ = 2.3, *P* = .1, η _p_^2^ = 0.079), although it did interact with genotype (F_[2,54]_ = 6.0, *P < *.01, η _p_^2^ = 0.18) such that both 1 and 3 mg/kg increased ASR in HIVtg rats only (F_[2,28]_ = 7.4, *P < *.01; Figure 3B). No main effects of sex (F_(1,27) < _0.001, *P* > .99, η _p_^2 < ^0.001) or genotype were observed on ASR (F_[1,27]_ = 0.9, *P* = .4, η _p_^2^ = 0.032), although a sex × genotype interaction was detected (F_(1,27)_ = 5.0, *P < *.05, η _p_^2^ = 0.16) whereby WT males exhibited greater ASR than WT females regardless of dose (F_[1,14]_ = 28.1, *P < *.001; η _p_^2^ = 0.67). No such effect of sex was observed among HIVtg rats (F_[1,13]_ = 1.2, *P* = .3; η _p_^2^ = 0.085). Secondary measures are reported in supplementary Results and presented in supplementary Table 1.

### CBD Assessment

**Figure 4.**
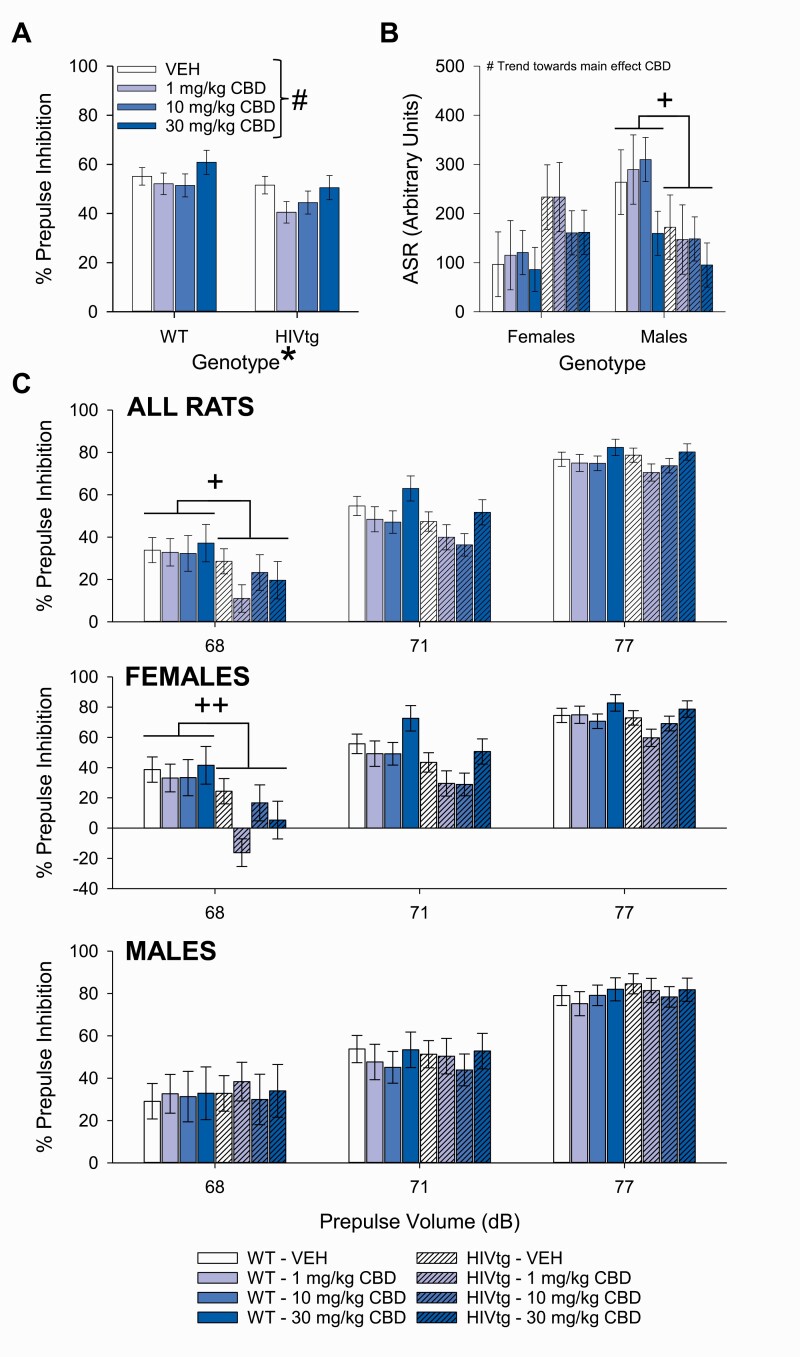
The effects of cannabidiol (CBD) on prepulse inhibition (PPI) of the acoustic startle response (ASR) in male and female HIV transgenic (HIVtg) and wild-type (WT) rats. A main effect of genotype was observed on PPI (averaged across prepulse intensities), whereby HIVtg rats demonstrated lower levels of sensorimotor gating than WT rats regardless of sex or CBD treatment. A non-significant trend of CBD was also observed on PPI regardless of genotype or sex (A). No main effects of genotype or sex were observed on ASR, although a sex × genotype interaction indicated an HIV-transgene-mediated reduction in males only. A non-significant trend of CBD was also observed regardless of any other variable (B). Post hoc analysis of a near-significant trend toward prepulse × genotype interaction revealed that HIVtg rats demonstrated reduced PPI relative to WT rats following prepulses of 68 dB only (C) (top); further analysis revealed that this reduction was driven by females (middle). ^+^*P*<.05; ^++^*P*<.01; *significant main effect; #non-significant trend toward main effect (*P*<.10). Data presented as mean ± SEM.

HIVtg rats demonstrated reduced PPI relative to WT rats regardless of CBD treatment (F_[1,28]_ = 4.3, *P < *.05, η _p_^2^ = 0.13; Figure 4A). A trend toward a main effect of CBD was observed on PPI (F_[3,84]_ = 2.5, *P* = .065, η _p_^2^ = 0.082; Figure 4A); pairwise comparisons revealed that 1 mg/kg CBD subtly lowered PPI relative to vehicle (*P* = .05) and that PPI was higher following 30 mg/kg CBD than 1 mg/kg (*P < *.05). A non-significant trend of sex indicated that females tended toward lower PPI than males (F_[1,28]_ = 2.9, *P* = .098, η _p_^2^ = 0.095), and a sex × genotype interaction (F_[1,28]_ = 6.0, *P < *.05, η _p_^2^ = 0.18) revealed an HIV transgene-mediated PPI reduction in females only (F_[1,14]_ = 10.1, *P < *.01, η _p_^2^ = 0.42). As in the THC assessment, PPI increased commensurately with prepulse level (F_[2,56]_ = 195.0, *P < *.001, η _p_^2^ = 0.87). Prepulse intensity tended to interact with genotype (F_[2,56]_ = 3.0, *P* = .059, η _p_^2^ = 0.096), with HIVtg rats exhibiting lower PPI than WT rats following 68-dB prepulses (F_[1,28]_ = 6.7, *P < *.05, η _p_^2^ = 0.19; Figure 4C, top); furthermore, a sex × genotype interaction was observed during 68-dB conditions (F_[1,28]_ = 9.2, *P < *.01, η _p_^2^ = 0.25) whereby female HIVtg rats exhibited lower PPI than female WT rats (F_[1,14]_ = 16.5, *P < *.01, η _p_^2^ = 0.54; Figure 4C, middle). Planned analysis of vehicle data alone revealed no effects of genotype (F_(1,28)_ = 0.5, *P* = .50, η _p_^2^ = 0.017), sex (F_(1,28)_ = 0.5, *P* = .50, η _p_^2^ = 0.017), or genotype × prepulse interaction (F_[2,56]_ = 0.8, *P* = .4, η _p_^2^ = 0.29) on PPI, and further planned analyses of the 2 genotypes individually revealed no main effects of CBD on either WT (F_[3,42]_ = 1.5, *P* = .2, η _p_^2^ = 0.095) or HIVtg rats (F_[3,42]_ = 1.5, *P* = .2, η _p_^2^ = 0.094).

A trend toward a main effect of CBD was observed on ASR (F_[3,84]_ = 2.7, *P* = .053, η _p_^2^ = 0.087; Figure 4B) whereby 30 mg/kg CBD lowered ASR relative to vehicle (*P < *.01) and 1 (*P < *.01) and 10 mg/kg CBD (*P < *.05). CBD did not interact with genotype (F_[3,84]_ = 0.8, *P* = .5, η _p_^2^ = 0.028) or sex (F_[3,84]_ = 0.7, *P* = .5, η _p_^2^ = 0.026) on this measure. No main effects of genotype (F_[1,28]_ = 0.06, *P* = .8, η _p_^2^ = 0.002) or sex (F_[1,28]_ = 1.1, *P* = .3, η _p_^2^ = 0.036) were observed on ASR; however, a significant interaction was detected (F_[1,28]_ = 5.1, *P < *.05, η _p_^2^ = 0.15) whereby male HIVtg rats exhibited lower ASR than male WT rats (F_[1,14]_ = 9.2, *P < *.01, η _p_^2^ = 0.40). Secondary measures are reported in supplementary Results and summarized in supplementary Table 2.

## Discussion

Consistent with our primary hypothesis and with findings in PWH (Minassian et al, 2013; Walter et al., 2021), HIVtg rats exhibited PPI deficits relative to WT controls. Our secondary hypothesis was also supported, as an altered sensitivity to THC was observed in HIVtg vs WT rats. The nature of these effects contradicted our final hypothesis, however, with THC impairing PPI in WT rats without affecting PPI in HIVtg rats. CBD did not significantly affect primary measures of sensorimotor gating in HIVtg or WT rats. The present findings support the use of the HIVtg rat as a model of aberrant neural processing in PWH and the premise that cannabinoids exert differential effects based on HIV status. These results also suggest that chronic exposure to HIV proteins may reduce sensitivity to the acute psychotomimetic effects of THC. This relative insensitivity suggests that PWH may (1) require higher levels of THC than healthy individuals in order to experience the same effects of cannabis, and/or (2) experience fewer or less severe negative effects associated with fronto-striatal impairment. Either of these possibilities may serve to partially explain the higher rates of overall and heavy cannabis use observed among PWH relative to the general population (Compton et al., 2016).

In contrast with previous findings (Moran et al., 2013b), planned analysis of vehicle data alone during THC testing (Figure 3A)—and a main effect of genotype across all doses of CBD (Figure 4A)—revealed a previously undetected general PPI deficit of the HIVtg rat. Critically, this deficit was driven by the 68-dB prepulse condition, as indicated by a significant prepulse × genotype interaction during the THC assessment (Figure 3C) and a similar near-significant trend during the CBD assessment (Figure 4C). In this condition, prepulses were only 3 dB louder than background noise levels (65 dB), indicating that the PPI deficit of the HIVtg rat is specific to prepulse intensities that are considerably lower than those utilized in previous study (i.e., 15 dB above background; Moran et al., 2013b). Further comparisons between present and extant findings are difficult, given that the present study utilized a different startle paradigm than that typically used to assess HIVtg rats. To wit, the present paradigm manipulated prepulse intensity, while previous studies manipulated inter-stimulus interval (ISI; e.g., Moran et al., 2012). While our decision to eschew ISI manipulation precluded investigation of the robust timing deficits of the HIVtg rat (McLaurin et al., 2019a), the present paradigm was chosen because it was deemed more likely to recreate the specific PPI deficit observed in HAND patients (Walter et al., 2021; Minassian et al., 2013). This judgment was made for 2 reasons. First, timing deficits have yet to be observed in HAND—the PPI deficit reported in PWH was identified using a startle paradigm that used prepulse intensity, not ISI duration, as a variable and that had previously revealed deficits in patients with obsessive-compulsive disorder and schizophrenia (Minassian et al., 2007; Ahmari et al., 2012). Second, this HIV-mediated PPI deficit was observed not as a sub-standard decrease in overall startle reactivity to prepulse-preceded stimuli (the units typically reported in HIVtg rat studies), but as a reduction in the percent decrement of the ASR to prepulse-paired vs unpaired stimuli—a deficit not observed in HIVtg rats tested in ISI-centric paradigms (Moran et al., 2013b). Given that our group had previously identified PPI deficits in rodent models of psychiatric disease following prepulses of only certain volumes (e.g., Geyer et al., 1993), we had reason to believe that manipulating this variable would reveal a similar prepulse-specific deficit in the HIVtg rat. Indeed, by including a range of prepulse intensities, the present study was able to detect a PPI deficit in the HIVtg rat of a type that is directly comparable with clinical findings.

HIV genotype did not independently affect ASR during pulse-alone trials during either the THC or CBD assessments, thus enabling the interpretation that the observed reduction in percent PPI was due to a genuine sensorimotor gating deficit rather than generally reduced startle reactivity to both prepulse-paired and unpaired pulses (Swerdlow et al., 2000). Previous studies of the HIVtg rat have reported mixed findings on this measure, however (i.e., ASR during a “0-msec inter-stimulus interval” condition). To wit, male and female HIVtg rats exhibited alternately higher (Moran et al., 2012, 2013b), lower (Moran et al., 2013a; McLaurin et al., 2017a, b, 2018c, 2019b), and similar (Moran et al., 2013a; Roscoe et al., 2014) startle reactivity to unpaired pulses relative to WT controls at various ages, although statistical analyses of this variable were not reported in several of these publications. Of greatest relevance to the present study, in which rats were 3.5 months old at time of THC assessment, are potentially conflicting findings in 3-, 4-, and 5-month-old male and female rats. At 3 (McLaurin et al., 2018c) and 4 months of age (Moran et al., 2013a), male and female HIVtg rats apparently exhibited reduced ASR relative to controls (no statistical analyses reported), while a different cohort of female HIVtg rats demonstrated significantly elevated startle reactivity at 5 months (Moran et al., 2013b). An explanation for this inconsistency may be a natural inter-individual (and possibly inter-cohort) neural heterogeneity within nuclei relevant to the startle response, as is revealed in the HIVtg medial prefrontal cortex (mPFC) by chronic psychostimulant self-administration (McLaurin et al., 2018b). While it cannot be determined whether this inter-individual cortical heterogeneity directly accounts for the variability in ASR, it is possible that similar variability exists in other centers linked to this reflex. Regardless of underlying mechanisms, however, it is apparent from the heterogeneity of extant reports of HIVtg ASR that the absence of effect reported herein is not out of line with the established phenotype of the HIVtg rat and does not indicate any gross deviation from the norm.

Contrary to our hypothesis, the PPI deficit of the HIVtg rat was not ameliorated by THC. Meanwhile, THC significantly lowered PPI in WT rats (Figure 3A), consistent with near-significant trends reported by the only 2 extant studies of acute subcutaneous THC of which we are aware (Hložek et al., 2017; Uttl et al., 2018). Critically, this reduction of PPI in WT rats confirms the biological activity of subcutaneous THC at the doses investigated, enabling the interpretation that chronic expression of HIV proteins reduced the sensitivity of HIVtg rats to otherwise PPI-disruptive doses of THC. Thus, it may be that PWH use cannabis at higher rates than the general population not only because of its therapeutic effects (e.g., antiemesis, anti-inflammation, etc.; de Jong et al., 2005; Manuzak et al., 2018), but because they require higher doses to receive the same subjective effects or, indeed, because they are less sensitive to the negative consequences that may be predicted by impaired PPI. Such a lack of deleterious side effects of THC would support its continued therapeutic use in this population.

THC meanwhile potentiated the ASR of HIVtg rats, but not WT rats, to unpaired pulses (Figure 3B). This lack of effect on WT rats is consistent with previous reports of acute subcutaneous THC failing to alter ASR at doses that arithmetically reduced PPI (Hložek et al., 2017); however, the effect on HIVtg rats is without precedent. Notably, no genotype-specific effects of THC were observed on ASR during the 5 trial startle periods at the beginning and end of the session (supplementary Table 1). The HIVtg ASR data can therefore be interpreted in 2 ways: (1) THC specifically affected ASR only during the middle of startle testing, altering the rate of intra-session habituation of HIVtg rats so subtly that both genotypes still displayed the same overall startle reactivity at the end of the session; or (2) the effect of THC on HIVtg ASR was too subtle to be observed within the narrow 5-trial windows and could only emerge across the 14 main session pulse-alone trials. Critically, either way, this effect verifies the biological activity of THC in HIVtg rats at the given doses and therefore helps validate the interpretation that the lack of effect on HIVtg PPI reflects a genotype-specific reduction in THC responsivity.

CBD did not exert any significant deleterious main effects on ASR or PPI in either WT or HIVtg rats, nor did it exert any PPI-restorative effects in HIVtg rats (Figure 4A–B). Near-significant trends of CBD were observed on these measures, however, with 30 mg/kg reducing ASR and 1 mg/kg reducing PPI. These findings are consistent with 1 of 2 extant studies of acute intraperitoneal CBD on rat startle, although the dose-response dynamics appear to be different; to wit, CBD reduced the ASR of Sprague-Dawley rats at 3 and 10 mg/kg and reduced PPI at 10 mg/kg (Gururajan et al., 2011). No such reductions to either measure were observed in Wistar rats, meanwhile (Levin et al., 2014). This minor discrepancy between past and present studies may reflect an influence of rat strain on the outcome of what is apparently a subtle and dose-dependent action of CBD on startle circuitry. Indeed, given that the trends of CBD on ASR and PPI detected by the present study were (1) not significant at the *P* <.05 level and (2) not observable at all following separation of genotypes and consequent reduction of sample sizes, it can be concluded that CBD does not exert meaningful effects on sensorimotor gating in either HIVtg or WT Fischer 344 rats (minor sex- and dose-dependent effects on HABIT1 ASR notwithstanding) (supplementary Table 2).

Although the underlying mechanisms of THC and CBD were not investigated, the neural circuitry mediating ASR and PPI has been sufficiently characterized to allow for speculation. The nucleus accumbens (NAc), for example, is a critical region in the regulation of sensorimotor gating (Swerdlow et al., 2001). The primary cell type of the NAc, the medium spiny neuron (MSN) (Kemp and Powell, 1971), integrates ascending dopaminergic input from the ventral tegmental area (VTA) with descending glutamatergic signals from forebrain and limbic structures (Swerdlow et al., 2001). In general, pharmacological manipulations that enhance NAc dopamine tone induce PPI deficits in rats (Swerdlow et al., 2001). Acute systemic THC increases extracellular dopamine concentration in the NAc shell (Chen et al., 1990; Tanda et al., 1997)—a potentially similar action to that of PPI-disruptive doses of intra-shell d-amphetamine (Wan and Swerdlow, 1996). Notably, this THC-mediated increase in accumbal dopamine is blocked by both systemic cannabinoid CB1 receptor (CB1R) blockade and opioid receptor antagonism in the VTA (Tanda et al., 1997), the primary source of NAc dopamine (Groenewegen et al., 1991). This latter finding, in combination with reports of systemic THC increasing firing rates of dopaminergic VTA neurons projecting to the NAc shell (Secci et al., 2019), suggests that (1) the VTA is likely a mediator of THC-induced NAc dopamine release, and (2) the effects of THC on mesoaccumbal dopamine transmission are not directly realized by CB1R binding on mesencephalic/striatal dopaminergic neurons. The role of mesoaccumbal CB1R activation in PPI disruption is likely nominal, as infusion of the full CB1R agonist WIN 55,212-2 into the VTA or the NAc does not induce PPI alterations (Wegener et al., 2008). PPI is, however, reduced when WIN 55,212-2 is infused into the ventral hippocampus and mPFC (Wegener et al., 2008), both of which significantly enhance VTA excitation (and ostensibly mesoaccumbal dopamine release; Kalivas et al., 1989) following THC administration (Loureiro et al., 2015; Hudson et al., 2019; Secci et al., 2019). The PPI-disruptive effect of THC in WT rats may therefore have been partially mediated by CB1R activation in the mPFC and/or ventral hippocampus enhancing VTA glutamate transmission, and thereby stimulating dopamine release in the NAc shell. Critically, MSNs of the HIVtg NAc core exhibit functionally reduced synaptic connectivity and consequently receive less dopaminergic input from the VTA (Roscoe et al., 2014; Javadi-Paydar et al., 2017; McLaurin et al., 2018c). Assuming that this synaptic dysfunction extends into the NAc shell, such neuropathology may have hindered any THC-mediated increase in mesoaccumbal dopamine transmission and thereby prevented any reduction of PPI. Importantly, both THC and HIV genotype independently produced mere 20% reductions of PPI relative to WT baseline—relatively minor effects vs those of other experimental manipulations, for example, apomorphine (Mansbach et al., 1988). This relative subtlety of drug and genotype effects suggests, respectively, (1) only modest alteration of PPI-relevant neurocircuitry by THC, and (2) sub-maximally impaired PPI in HIVtg rats at baseline. It is therefore unlikely that the absence of drug-induced PPI alteration in HIVtg rats was due to a floor effect.

No 3-way interactions were observed between THC, genotype, and sex on PPI. This lack of sex-specificity is somewhat surprising given that THC has been reported to differentially affect female vs male rats, with females being more susceptible to THC-induced hypothermia (Borgen et al., 1973) and antinociception (Tseng and Craft, 2001). Of greater relevance to the present study are sex differences in MSN morphology among HIVtg rats. While accumbal synaptic connectivity is reduced in both male and female rats, the specific morphological alterations that produce this reduction vary across sexes; males exhibit generally reduced dendritic spine volume (McLaurin et al., 2018c), while a population shift toward reduced spine length (Roscoe et al., 2014; Javadi-Paydar et al., 2017), dendritic branching, and arbor complexity (McLaurin et al., 2018c) is observed in females. Given the tendency for dopaminergic vs glutamatergic afferents to synapse on MSNs at specific points along the spines (i.e., neck vs head; Freund et al., 1984; Zahm, 1992), these morphological differences may be predicted to give rise to different ratios of dopaminergic vs glutamatergic innervation of MSNs across sexes, possibly resulting in differential responsivity to pharmacological manipulation. Unfortunately, since the present study made no attempt to quantify MSN dendritic morphology, it cannot be determined whether any such differences in accumbal connectivity between genotypes or sexes had any influence on THC responsivity. It should be noted, however, that significant sex × genotype interactions did eventually emerge in the present cohort of rats, with HIVtg males demonstrating lower ASR than WT males during the CBD assessment. Meanwhile, the genotype-mediated PPI deficit persisted only in females, possibly the result of a floor effect on male startle reactivity driven by an inter-time point reduction in overall ASR. This emergence of sex effects may reflect a sex-specific progression of HIV-mediated neuronal insult during the 4-month washout period, highlighting the need for evaluation of the effects of THC in older HIVtg rats.

The 4-month washout period must also be considered in the context of the progression of clinical pathologies associated with the HIV transgene. Critically, while the original HIVtg line developed by Reid and colleagues (2001) presented with a syndrome of severe dermatological, cardiac, renal, and neurological pathology beginning at 5–9 months of age, the derivation of HIVtg rat utilized by the present study expresses a far more moderate phenotype. Consistent with previous characterizations of the contemporary HIVtg rat (in which the transgene is restricted to chromosome 9) (Peng et al., 2010; Moran et al., 2013a; McLaurin et al., 2018a), the cohort assessed herein demonstrated no overt signs of wasting or accelerated mortality between 3 and 8 months of age, and cataracts were present throughout the study. The absence of main and interactive effects of genotype on inter-trial movement during both the THC and CBD assessments (“no stimulus”; supplementary Tables 1 and 2) indicated a minimal contribution of any genotype-mediated motor alteration to PPI. Indeed, previous longitudinal assessment of HIVtg locomotor activity revealed no gross motor impairment between 90 and 480 days of age, although PPI assessment of these animals identified a change in optimal prepulse-pulse interval between 90 and 240 days of age (McLaurin et al., 2018a). Interestingly, within-subjects analysis of viral mRNA levels in male HIVtg rats at 2–3 and 10–11 months of age revealed a shift toward higher striatal and prefrontocortical HIV protein expression over time (Peng et al., 2010). This increase in viral protein expression in PPI-relevant brain regions may partially account for the inter-time-point variability observed in past and present assessments; however, it is difficult to directly attribute the emergence of sex effects to this phenomenon as age-specific viral expression has yet to be evaluated in female HIVtg rats. Direct comparison of the above THC and CBD effects is complicated as well, as CB1R expression appears to be similarly age dependent in the rat (Liu et al., 2003). Furthermore, the HIV protein Tat reduces CB1R function in rat hippocampal cultures (Wu and Thayer, 2020); this action may conceivably impact other PPI-regulatory nuclei in vivo and may potentiate over time with increased expression. This likelihood of significant inter-time point differences in HIVtg neuropathology necessitates interpretation of the results of the 2 drug assessments separately and in the context of age. Caution must be taken in generalizing the present findings across time points.

Given that group-level PPI deficits were first observed among only those PWH diagnosed with HAND (Minassian et al., 2013) and that lower baseline PPI correlated to poorer learning and memory in HIV^+^ men (Walter et al., 2021), PPI deficits may be an indicator of cognitively relevant frontal system dysfunction in HIV. Following this interpretation, the present finding that acute THC does not exacerbate the PPI deficit of HIVtg rats suggests that acute cannabis use may not produce the same level of cognitive impairment in HAND patients as in healthy subjects or even PWH who have not yet developed significant frontal systems damage. This interpretation is, however, severely limited by 3 main factors, all of which merit consideration in future studies of cannabinoids and HIV. The first limitation of the present study is that acute subcutaneous and/or i.p. THC and/or CBD cannot be expected to produce effects representative of those of conventional human cannabis use, which is typically characterized by repeated smoking, vaping, or ingestion. For example, repeated administration of THC creates long-term changes to dendritic spine morphology in the NAc (Kolb et al., 2006, 2018), which may be relevant to PPI in the context of the mechanisms described above. Such chronicity may account for the more severe effects of moderate-to-heavy cannabis consumption on learning and memory in PWH vs healthy individuals (Thames et al., 2016), which would not have been predicted by the results of the present study. The pharmacokinetic differences between routes of THC/CBD administration must also be taken into account, given that subcutaneous administration produces far less rapid increases in brain and serum cannabinoid levels than pulmonary administration in rats and lower peak brain cannabinoid levels than oral administration (Hložek et al., 2017). Secondly, the present study is limited in that it only characterized the effects of individual cannabinoids on PPI. *Cannabis sativa* contains more than 60 unique cannabinoids (Mechoulam, 2005) that may potentially interact with each other in a manner relevant to sensorimotor gating. Indeed, such an interaction has been reported between subcutaneous THC and CBD in rats, whereby their co-administration mitigated their individual PPI-disruptive effects (Hložek et al., 2017). Finally, it is important to note that none of the rats used in the present study were maintained on cART, the primary medication regimen for the suppression of HIV. It is currently unknown whether cART affects sensorimotor gating, although chronic administration does induce cognitive impairments in healthy mice (Pistell et al., 2010). Each of these limitations—treatment chronicity/route, potential inter-cannabinoid interactions, and cART effects—need to be addressed when extrapolating the present results to the potential consequences of cannabis use by PWH.

In conclusion, the present study identified a PPI deficit in the HIVtg rat that is (1) qualitatively similar to that observed among PWH and (2) not exacerbated by acute administration of otherwise PPI-disruptive doses of THC at approximately 3.5 months of age, or by CBD at approximately 8 months. Given that PPI deficits may be associated with cognitive decline in the HIV disease state (Minassian et al., 2013; Walter et al., 2021), these results may be tentatively interpreted as indicative of an HIV-conferred resistance to the deleterious effects of THC on overlapping cognition- and PPI-relevant neurocircuitry. Future studies are needed to validate this conclusion, however, which may include clinical investigation of the interactions between HIV and cannabis on PPI and cognition as well as between HIV and the subjective and therapeutic effects of THC.

## Supplementary Materials

Supplementary data are available at *International Journal of Neuropsychopharmacology (IJNPPY)* online.

Supplementary Table S1. Secondary measures from THC assessment. Bolded text denotes *P < *.05; refer to main text for post hoc analyses of significant interactions. “HABIT1” and “HABIT2” values report the average ASR during the first and last 5 pulse-alone trials, respectively; units are arbitrary. Overall ASR during HABIT1 and HABIT2 periods significantly differed at the *P < *.001 level. “No Stimulus” values report overall movement during inter-trial intervals; units are arbitrary.

Supplementary Table S2. Secondary measures from CBD assessment. Bolded text denotes *P < *.05; refer to main text for post hoc analyses of significant interactions. “HABIT1” and “HABIT2” values report the average acoustic startle response during the first and last 5 pulse-alone trials, respectively; units are arbitrary. Overall ASR during HABIT1 and HABIT2 periods significantly differed at the *P < *.001 level. “No stimulus” values report overall movement during inter-trial intervals; units are arbitrary.

pyab053_suppl_Supplementary_Materials

pyab053_suppl_Supplementary_Table_1

pyab053_suppl_Supplementary_Table_2

## Interest Statement

M.A. Geyer holds an equity interest in San Diego Instruments. The other authors report no conflicts of interest.
