# Supplementary material for: HIV Transgenic Rats Demonstrate Impaired Sensorimotor Gating But Are Insensitive to Cannabinoid (Δ9-Tetrahydrocannabinol)-Induced Deficits
Source: Int J Neuropsychopharmacol. 2021 Aug 2;24(11):894–906. doi: 10.1093/ijnp/pyab053 (PMC8598295; doi:10.1093/ijnp/pyab053)
Supplement: pyab053_suppl_Supplementary_Materials [file pyab053_suppl_supplementary_materials.docx]

**Secondary Outcome Measures of the Acoustic Startle Paradigm**

The acoustic startle paradigm included three secondary outcome measures:

1. HABIT1- and HABIT2 ASR ­­– the ASR elicited by unpaired 120 dB prepulses during the first and last 5 trials of a given startle session, respectively. Reported in arbitrary units.
2. Habituation of startle response within testing sessions, calculated from HABIT1 and HABIT2 ASR data. Reported as % habituation.
3. Activity level during no-stimulus conditions, i.e., during inter-trial intervals.

**Statistical Analysis**

ASR data recorded during the HABIT1 and HABIT2 phases of both assessments were analyzed via repeated measures ANOVAs, using drug dose (THC or CBD) and habituation period (i.e., HABIT1 versus HABIT2) as within-subjects factors and sex and genotype as between-subjects factors. Percent habituation and movement during non-stimulus conditions were both analyzed via three-factor ANOVAs, with drug dose as a within-subjects factor, and sex and genotype as between-subjects factors.

**Results**

*THC Assessment*

Data from secondary measures of the startle paradigm are presented in Supplemental Table S1. THC did not affect ASR during HABIT1 [F<1, n.s.], although 3 mg/kg THC non-significantly increased ASR during HABIT2 relative to vehicle [F(2,54)=2.9, p=0.062]. A main effect of sex was observed during HABIT1 [F(1,27)=6.4, p<0.05], with males demonstrating greater ASR than females. No interactions between THC, sex, or genotype were observed during either HABIT1 or HABIT2, although a sex × genotype interaction almost reached significance during HABIT2 [F(1,27)=4.1, p=0.053]. ASR was higher during HABIT1 than in HABIT2 across groups [F(1,54)=89.6, p<0.001]. Males underwent higher levels of habituation within the testing session than females [F(1,27)=9.1, p<0.01], and HIVtg rats tended toward greater habituation than WT rats [F(1,27)=3.9, p=0.059]; this latter trend, however, failed to reach significance. Male rats also exhibited higher levels of movement during inter-trial intervals than females [F(1,27)=5.3, p<0.05]. No main or interactive effects of THC or genotype were observed on this latter measure.

*CBD Assessment*

Data from secondary measures are presented in Supplemental Table S2. ASR during HABIT1 was increased relative to vehicle by 10 mg/kg CBD, and decreased by 30 mg/kg CBD [F(3,84)=5.8, p<0.01]. These effects were sex-dependent, however [F(3,84)=3.3, p<0.05] – HABIT1 ASR was increased by 10 mg/kg CBD in females [F(3,45)=4.2, p<0.05], and decreased by 30 mg/kg CBD in males [F(3,45)=4.8, p<0.01]. Overall, male rats exhibited higher HABIT1 ASR than females [F(1,28)=5.5, p<0.05], although a sex × genotype interaction [F(1,28)=6.1, p<0.05] revealed that this effect was driven by the WT group, within which males exhibited higher ASR than females [F(1,14)=17.8, p<0.01]. 30 mg/kg CBD decreased ASR during HABIT2 regardless of genotype [F(3,84)=2.8, p<0.05]. HIVtg rats demonstrated lower ASR during HABIT2 than WT rats [F(1,28)=5.9, p<0.05], although a sex × genotype interaction [F(1,28)=5.7, p<0.05] indicates that this effect was driven by male WT rats exhibiting nearly double the ASR of every other group. As in the THC assessment, ASR during HABIT1 was higher than ASR during HABIT2 [F(1,28)=57.7, p<0.001]. Significant effects of sex [F(1,28)=10.0, p<0.01], genotype [F(1,28)=18.8, p<0.01], and sex × genotype interaction [F(1,28)=6.2, p<0.05] on % habituation indicate that males underwent greater habituation than females, and that HIVtg rats underwent greater habituation than WT rats. These effects were likely driven by the female WT group, which exhibited % habituation values approximately half those of every other group. No main or interactive effects of CBD, genotype, or sex, were observed on movement between trials [F’s<3, n.s.].
