# Supplementary material for: HIV Transgenic Rats Demonstrate Impaired Sensorimotor Gating But Are Insensitive to Cannabinoid (Δ9-Tetrahydrocannabinol)-Induced Deficits
Source: Int J Neuropsychopharmacol. 2021 Aug 2;24(11):894–906. doi: 10.1093/ijnp/pyab053 (PMC8598295; doi:10.1093/ijnp/pyab053)
Supplement: pyab053_suppl_Supplementary_Table_1 [file pyab053_suppl_supplementary_table_1.docx]

**Table 1.** Secondary Measures from THC Assessment.

| **Measure** | **THC Dose** | **Mean (_­_±SEM)** | | | | **THC** | **Gene** | **Sex** | **THC × Gene** | **Sex × Gene** |
| --- | --- | --- | --- | --- | --- | --- | --- | --- | --- | --- |
|  |  | **WT** | | **HIVtg** | | **F_(2,54)_, *p*** | **F_(1,27)_, *p*** | **F_(1,27)_, *p*** | **F_(2,54)_, *p*** | **F_(1,27)_, *p*** |
|  |  | **Female** | **Male** | **Female** | **Male** |  |  |  |  |  |
| **HABIT1** | **VEHICLE** | 197.7 (95.6) | 666.6 (95.6) | 324.8 (102.3) | 488.9 (95.6) | <1, ns | <1, ns | **6.4, *p*<0.05** | 2.0, ns | 2.5, ns |
|  | **1 mg/kg** | 231.2 (105.2) | 595.4 (105.2) | 386.3 (112.4) | 501.4 (105.2) |  |  |  |  |  |
|  | **3 mg/kg** | 195.6 (110.6) | 470.3 (110.6) | 506.4 (118.2) | 483.8 (110.6) |  |  |  |  |  |
| **HABIT2** | **VEHICLE** | 79.5 (26.0) | 150.9 (26.0) | 130.0 (27.8) | 53.4 (26.0) | 2.9, *p*=0.062 | <1, ns | <1, ns | <1, ns | 4.1, *p*=0.053 |
|  | **1 mg/kg** | 105.3 (75.5) | 171.8 (75.5) | 240.7 (80.7) | 89.5 (75.5) |  |  |  |  |  |
|  | **3 mg/kg** | 103.8 (61.9) | 197.3 (61.9) | 260.0 (66.1) | 118.2 (61.9) |  |  |  |  |  |
| **% Habituation** | **VEHICLE** | 46.6 (12.7) | 73.9 (12.7) | 57.8 (13.6) | 88.6 (12.7) | 1.2, ns | 3.9, *p*=0.059 | **9.1, *p*<0.01** | <1, ns | <1, ns |
|  | **1 mg/kg** | 53.7 (9.2) | 60.9 (9.2) | 57.6 (9.8) | 80.2 (9.2) |  |  |  |  |  |
|  | **3 mg/kg** | 41.5 (7.9) | 55.6 (7.9) | 53.7 (8.5) | 74.7 (7.9) |  |  |  |  |  |
| **No Stimulus** | **VEHICLE** | 0.29 (0.14) | 0.80 (0.14) | 0.23 (0.15) | 0.43 (0.14) | 2.0, ns | 1.6, ns | **5.3, *p*<0.05** | <1, ns | 2.2, ns |
|  | **1 mg/kg** | 0.42 (0.28) | 0.86 (0.28) | 0.41 (0.29) | 0.62 (0.28) |  |  |  |  |  |
|  | **3 mg/kg** | 0.09 (0.15) | 0.74 (0.15) | 0.26 (0.16) | 0.21 (0.15) |  |  |  |  |  |
