# Supplementary material for: HIV Transgenic Rats Demonstrate Impaired Sensorimotor Gating But Are Insensitive to Cannabinoid (Δ9-Tetrahydrocannabinol)-Induced Deficits
Source: Int J Neuropsychopharmacol. 2021 Aug 2;24(11):894–906. doi: 10.1093/ijnp/pyab053 (PMC8598295; doi:10.1093/ijnp/pyab053)
Supplement: pyab053_suppl_Supplementary_Table_2 [file pyab053_suppl_supplementary_table_2.docx]

**Table 2.** Secondary Measures from CBD Assessment.

| **Measure** | **CBD Dose** | **Mean (_­_±SEM)** | | | | **CBD** | **Gene** | **Sex** | **CBD × Gene** | **Sex × Gene** |
| --- | --- | --- | --- | --- | --- | --- | --- | --- | --- | --- |
|  |  | **WT** | | **HIVtg** | | **F_(3,84)_, *p*** | **F_(1,28)_, *p*** | **F_(1,28)_, *p*** | **F_(3,84)_, *p*** | **F_(1,28)_, *p*** |
|  |  | **Female** | **Male** | **Female** | **Male** |  |  |  |  |  |
| **HABIT1** | **VEHICLE** | 132.7 (79.0) | 670.0 (79.0) | 330.5 (79.0) | 391.4 (79.0) | **5.9, *p*<0.01** | <1, ns | **5.5, *p*<0.05** | <1, ns | **6.1, *p*<0.05** |
|  | **1 mg/kg** | 150.5 (109.7) | 690.0 (109.7) | 393.9 (109.7) | 448.8 (109.7) |  |  |  |  |  |
|  | **10 mg/kg** | 273.3 (120.6) | 665.7 (120.6) | 509.0 (120.6) | 378.3 (120.6) |  |  |  |  |  |
|  | **30 mg/kg** | 136.0 (93.0) | 379.4 (93.0) | 347.3 (93.0) | 322.0 (93.0) |  |  |  |  |  |
| **HABIT2** | **VEHICLE** | 88.6 (31.5) | 162.6 (31.5) | 109.0 (31.5) | 64.5 (31.5) | **2.8, *p*<0.05** | **5.9, *p*<0.05** | 2.2, ns | <1, ns | **5.7, *p*<0.05** |
|  | **1 mg/kg** | 92.7 (23.3) | 142.5 (23.3) | 90.9 (23.3) | 63.2 (23.3) |  |  |  |  |  |
|  | **10 mg/kg** | 74.6 (21.4) | 190.1 (21.4) | 59.8 (21.4) | 73.8 (21.4) |  |  |  |  |  |
|  | **30 mg/kg** | 62.8 (22.5) | 110.6 (22.5) | 56.3 (22.5) | 47.6 (22.5) |  |  |  |  |  |
| **% Habituation** | **VEHICLE** | 26.3 (8.1) | 71.6 (8.1) | 71.2 (8.1) | 81.2 (8.1) | <1, ns | **18.8, *p*<0.01** | **10.0, *p*<0.01** | 1.0, ns | **6.2, *p*<0.05** |
|  | **1 mg/kg** | 34.7 (6.9) | 77.4 (6.9) | 71.6 (6.9) | 85.5 (6.9) |  |  |  |  |  |
|  | **10 mg/kg** | 53.8 (11.4) | 60.1 (11.4) | 82.2 (11.4) | 70.7 (11.4) |  |  |  |  |  |
|  | **30 mg/kg** | 24.9 (10.7) | 71.5 (10.7) | 84.5 (10.7) | 88.9 (10.7) |  |  |  |  |  |
| **No Stimulus** | **VEHICLE** | 0.33 (0.36) | 0.86 (0.36) | 0.59 (0.36) | 0.63 (0.36) | 1.7, ns | <1, ns | 1.9, ns | <1, ns | 2.6, ns |
|  | **1 mg/kg** | 0.35 (0.53) | 1.75 (0.53) | 0.71 (0.53) | 0.42 (0.53) |  |  |  |  |  |
|  | **10 mg/kg** | 0.33 (0.33) | 0.83 (0.33) | 0.57 (0.33) | 0.46 (0.33) |  |  |  |  |  |
|  | **30 mg/kg** | 0.04 (0.35) | 0.30 (0.35) | 0.04 (0.35) | 0.79 (0.35) |  |  |  |  |  |
